# Antifungal Paper Based on a Polyborneolacrylate Coating

**DOI:** 10.3390/polym10040448

**Published:** 2018-04-17

**Authors:** Jiangqi Xu, Yujia Bai, Meijiao Wan, Yanhui Liu, Lei Tao, Xing Wang

**Affiliations:** 1 Beijing Advanced Innovation Center for Soft Matter Science and Engineering, Beijing Laboratory of Biomedical Materials, College of Life Science and Technology, Beijing University of Chemical Technology, Beijing 100029, China; 13121049677@163.com (J.X.); 18811406802@163.com (Y.B.); 15624977166@163.com (M.W.); liuyh@mail.buct.edu.cn (Y.L.); 2 The Key Laboratory of Bioorganic Phosphorus Chemistry & Chemical Biology (Ministry of Education), Department of Chemistry, Tsinghua University, Beijing 100084, China; leitao@mail.tsinghua.edu.cn

**Keywords:** antifungal, coating, paper products, polyborneolacrylate, surface stereochemistry

## Abstract

Paper documents and products are very susceptible to microbial contamination and damage. Fungi are mainly responsible for those biodeterioration processes. Traditional microbicidal strategies constitute a serious health risk even when microbes are dead. Ideal methods should not be toxic to humans and should have no adverse effects on paper, but should own a broad spectrum, good chemical stability and low cost. In this work, we utilize an advanced antimicrobial strategy of surface stereochemistry by applying a coating of a shallow layer of polyborneolacrylate (PBA), resulting in the desired antifungal performance. The PBA-coated paper is challenged with the most common air-borne fungi growing on paper, *Aspergillus niger* and *Penicillium* sp. Ten percent by weight of the coating concentration or a 19-μm infiltration of PBA is sufficient to keep the paper spotless. The PBA coating also exhibits significant inhibition of spores’ germination. After PBA coating, both physicochemical properties (paper whiteness, pH, mechanical strength) and inking performance display only slight changes, which are acceptable for general utilization. This PBA coating method is nontoxic, rapid and cost-effective, thus demonstrating great potential for applications in paper products.

## 1. Introduction

Paper documents and products are very common in our daily life. However, they are very susceptible to microbial contamination and damage under inadequate conservation conditions due to their organic composition and hygroscopicity [1]. Microorganisms, fungi in particular, are mainly responsible for paper deterioration because of their cellulolytic activity [2]. The activity of fungi on paper can produce several types of damage, such as different colored stains, discoloration of pigments or inks and changes in the chemical and physical properties of paper [3], harm human health far more than we think, even when the fungi are dead [4,5]. Unfortunately, air is the most common medium for the dispersal of tiny fungal spores and hyphal filaments, which fall on the surface of paper and may lead to possible degradation once they meet a suitable environment [6], thus making it a great problem for paper conservation and safe use.

Until now, several methods have been used to prevent and stop fungal deterioration of paper products. Physical methods include dehydration, gamma irradiation, low-oxygen environments, ultraviolet radiation, and extreme temperature treatment [7]. However, it is difficult to place the paper products in a proper environmental condition at all times. The physical methods do not have a long-term action, since their microbicidal action is only immediate. Chemical microbicides can be added to paper by directly coating or incorporation into cellulose pulp during the production process. Various types of antifungal agents such as azole antifungals [8], phenol derivatives [9], silver and zinc oxide composite [10,11], titanium dioxide [12], quaternary ammonium compounds [13], calcium propionate and parabens [1,3,14] have been reported to produce excellent antifungal activity. However, all existent antifungal methods have strengths and weaknesses: most of the methods mentioned above have potential toxicity to humans and the environment, which make them unsatisfactory for long-term usage [15,16,17,18]. Meanwhile, traditional antifungal methods using antibiotic and inorganic metal may increase the risks of developing drug resistance [19,20]. Therefore, the development of non-toxic and safer antifungal strategies to treat fungal biodeterioration for paper products is still an important task that still remains a challenge.

Chiral material interfaces have a great influence on cells’ adhesion and proteins’ adsorption [21,22], which inspired us to further develop antimicrobial materials or surfaces by utilizing the ubiquitous “chiral taste” of microbes. Recently, our group has successfully developed a series of borneol-based polymers [23,24] where polyborneolacrylate (PBA) showed unique antibacterial adhesion properties. They dramatically reduced bacterial attachment and biofilm formation. Importantly, PBA polymers are noncytotoxic and can be easily synthesized. Therefore, in this paper, we present PBA polymers as antifungal coatings by applying them to paper products through the spraying method. The antifungal effect of PBA coating was evaluated with the aim of selecting the optimal conditions, the lowest concentration of PBA solution with the best antifungal efficiency. The antifungal activity is challenged with two selected fungi, *Aspergillus niger* and *Penicillium* sp., which are air-borne and the most common fungi growing on paper [25]. In addition, the related changes in the paper’s physicochemical properties and inking performance are assessed.

## 2. Experimental

### 2.1. Materials

Common printer paper made of long fiber wood pulp (70 g m^−2^) was obtained from Deli Stationery Company (Ningbo, China). Isobornyl acrylate (BA, 93%) was purchased from Sigma-Aldrich Company (Dorset, UK). Chromatographic-grade methanol (CH_3_OH, 99.9%) and dichloromethane (CH_2_Cl_2_, 99%) were obtained from Tokyo Chemical Industry (TCI) (Tokyo, Japan). Malt extract agar was purchased from Aladdin (Shanghai, China). *Aspergillus niger* was obtained from CICC (Beijing, China) (CICC No. 2364), and *Penicillium* sp. was saved in our lab.

### 2.2. Preparation of PBA

Polymerization was carried out in a penicillin bottle according to the reported method [23]. First, BA monomers were dissolved in degassed methanol (*v/v* = 1:1). Then, 1% of ammonium persulfate (0.1 g·mL^−1^ of water solution) was added into the solution as an initiator. The reaction was maintained at 70 °C for 4 h. Finally, the product was further purified by alternate treatments with dichloromethane (dissolution) and methanol (precipitation). The product obtained was a white solid with a yield above 90%.

### 2.3. PBA Coating on Paper

The obtained PBA was dissolved in dichloromethane with concentrations of 0, 5, 10 and 15 wt % (0 wt % of PBA was the blank control with only dichloromethane), respectively. After autoclave sterilization, each sample of the printer paper (10 cm^2^) was uniformly coated with the polymer solution (0.5 mL) mentioned above via the spray method. A customized glass spray bottle (10 mL) was used for spraying with a working distance of 10 cm. Then, the sample was dried at room temperature overnight for solvent evaporation. Finally, the coated paper was cut into round (diameter of 1.5 cm) or square (1.5 × 1.5 cm^2^) sheets for further antifungal evaluation.

### 2.4. Characterization

Scanning electron microscopy (SEM, JSM-7800F, JEOL, Tokyo, Japan) was used to observe the morphologies of the paper samples. For the SEM observation of the sample cross-section, the paper samples were snap frozen rapidly in liquid nitrogen. The surface C and O contents of the samples were determined by energy-dispersive spectroscopy (EDS, S-4700 Hitachi, Tokyo, Japan). X-ray photoelectron spectroscopy (XPS, Thermo Fisher Scientific, Waltham, MA, USA) was used to study changes in surface functional groups after being coated. Fourier-transform infrared (FT-IR) spectroscopy was performed using a Shimadzu IRAffinity-1 spectrometer (Beijing, China) at wavelengths of 500–4000 cm^−1^. Attenuated total reflection Fourier transform infrared spectroscopy (ATR-FTIR, Perkin-Elmer Spectrum 100 spectrometer, Waltham, MA, USA) was used to analyze the surface chemical structure and composition.

### 2.5. Antifungal Colonization Test

The antifungal activity test was carried out according to the reported method [26]. *A. niger* and *Penicillium* sp. were used in this test. The fungus was cultivated on malt extract agar medium and incubated at 30 °C for 4 days according to the streak-plate method. Following incubation, cells were scraped from the nutrient agar by an inoculating loop and mixed with 2 mL of 0.9% saline by a vortex mixer until the cells were homogeneously dispersed. The concentration of spore suspension for each fungal species was determined with a hemocytometer and adjusted to 10^8^ spores mL^−1^ for the antifungal experiments.

Before the antifungal colonization test, PBA-coated paper sheets (diameter of 1.5 cm) with a concentration of 0, 5, 10 and 15% were disinfected under UV irradiation for 30 min. Then, four samples were fixed onto malt extract agar medium (diameter of 9 cm) with sterile tweezers following the sequence. Then, 10 µL of fungal suspension were dropped on the center of the plate and thermostatic cultured at 30 °C in an incubator. Fungal growth at different periods was observed and recorded with a digital camera. For this test, each experiment was repeated at least three times.

To detect the adhesion details, SEM was used to study the morphologies of *A. niger* on those papers. After 8 days of incubation, samples were immobilized with 2.5% glutaraldehyde for 2 h at 4 °C. Fungal cells were dehydrated by 50, 60, 70, 80, 90 and 100% ethanol for 20 min, respectively, and then neutralized with isoamyl acetate for 1 h. Finally, the samples were freeze-dried for 12 h prior to the SEM observation.

### 2.6. Spore Germination Test

Two models of the spore germination were designed in petri dishes to simulate the practical condition in which paper was contaminated during usage and storage. The spore suspension with a concentration of 10^8^ spores/mL was obtained according to the method above. Then, 100 µL of fungal suspension were dispersed uniformly on the surface of agar medium and incubated at 30 °C for 4 days. The spores grown on the surface of materials were used to mimic the air-borne Model-I; and the spore suspension coated on the surface of the agar medium was used to mimic a humid environment as Model-II. In the first model, the blank paper and the PBA-coated paper (10% of PBA) were fixed on malt extract agar medium following UV sterilization. Then, the spores were collected with an area of 0.5 cm^2^ from the agar medium mentioned above by an inoculating loop and dropped evenly onto each sample’s surface and thermostatically cultured at 30 °C in an incubator. In the second model, 100 µL of fungal suspension were dispersed uniformly on the surface of agar medium. Then, the blank paper and the PBA-coated paper (10%) were put onto the plates and cultured at 30 °C in an incubator. Fungal growth at different periods was observed and recorded with a digital camera. Antifungal capability was quantified by the measurement of the spore’s colonized areas. For this test, each experiment was repeated at least three times.

### 2.7. Physicochemical Properties Tests

Changes in the paper’s physicochemical properties after being coated with 5, 10 and 15% of PBA were evaluated in terms of brightness, water contact angle, pH and tensile strength. The changes of brightness were detected by an SC-80C automatic chromometer (Kang Guang Instrument Co., Ltd., Beijing, China) according to ISO 2470. The ISO brightness was obtained using the following equation:

Wr = 0.925 × Z + 1.16

where Wr represents ISO brightness and Z is the measured tristimulus value of the sample.

Water contact angle (CA) measurement was performed on a JC2000D3 (Zhongyi Kexin Technology Co., Ltd., Beijing, China) at room temperature. Deionized water was used here. The hydrogen ion concentration (pH) of paper extracts was measured by a cold extraction method according to GB/T 1545.2-2003 with a pH meter (Mettler-Toledo S470, Zurich, Switzerland). Tensile strength was determined with a testing machine (MTS systems Co., Ltd., Shanghai, China) at a crosshead speed of 20 mm/min with a 15-mm width for all the samples according to GB/T 12914-2008. At least ten identical specimens were tested for each sample, and their average mechanical properties were reported.

### 2.8. Inking Performance Test

The blank control and the PBA-coated papers (5, 10 and 15% of PBA) were used for this test. The letters “BUCT” were marked on the surface of the paper by ink-jet printing with a printer (HP LaserJet Pro MFP M225–M226, Shanghai, China) and handwriting with a marker pen (MG 2130, Shanghai, China). Then, the changes in writing clarity were observed by visual observation for four months and recorded with a digital camera. Colorimetric measurement was also conducted to evaluate the inking performance of the PBA-coated papers, according to the CIE L* a* b* Color System with Data Color International-Micro flash, using D65 illuminant. An ink jet printer (the same as mentioned above) and ink (HP 88A, Shanghai, China) were used in this test. The lightness difference (ΔL*), the red-green difference (Δa*) and the yellow-blue difference (Δb*) are calculated according to the following formula:

ΔL* = L* − L_t_*, Δa* = a* − a_t_*, Δb* = b* − b_t_*

where L*, a* and b* are the test values of paper after coating and L_t_*, a_t_* and b_t_* are the test values of blank paper. The total color difference, ΔE*, is obtained according to the following formula:

$$\Delta E*\;=\;\sqrt{{\left(\Delta L*\right)}^{2}+{\left(\Delta a*\right)}^{2}+{\left(\Delta b*\right)}^{2}}$$

## 3. Results and Discussion

### 3.1. Morphology of PBA-Coated Paper

**Figure 1:**
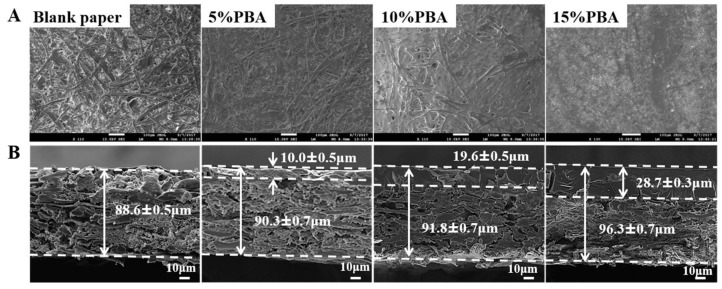
(**A**) SEM images of the blank control paper and papers coated with 5, 10 and 15% of PBA. (**B**) SEM images of the cross-sections: the blank control paper and papers coated with 5, 10 and 15% of PBA.

This PBA coating method was simple and efficient. PBA was dissolved in dichloromethane and uniformly sprayed onto paper. Then, PBA was attached to the paper with solvent evaporation. In fact, this procedure can mimic the printing process. Figure 1A shows the SEM images of the uniform PBA coating film on papers. With the increasing concentration of PBA, the thickness of the film increased and the texture of the paper fiber became less visible. SEM was also used to provide direct observation of the coating thickness. The cross-section images of the blank control paper and paper coated with 5, 10 and 15% of PBA are shown in Figure 1B. For the blank control paper, the thickness was approximately 88.6 μm. After coating with 5, 10 and 15% of PBA, polymer penetration layers were observed clearly, and the thickness of the layers was ~10.0, ~19.6 and ~28.7 μm, respectively. When the concentration was up to 15%, the total thickness was increased slightly (approximately 96.3 μm in Figure 1B). Otherwise, 5 and 10% of PBA coating did not change the thickness of the original paper according to the data.

### 3.2. Characterization of PBA-Coated Paper

**Figure 2:**
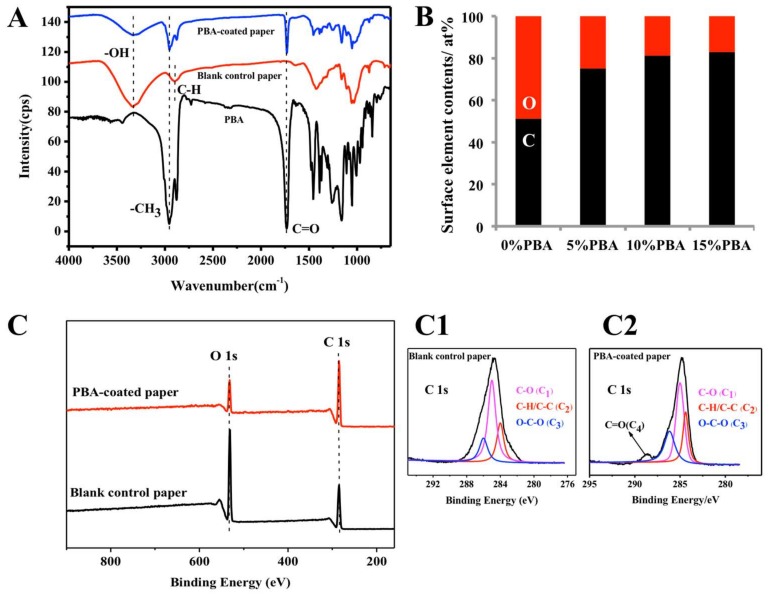
(**A**) ATR-FTIR spectra of the PBA-coated paper (10%) (blue line) and the blank control paper (red line), as well as the FTIR spectrum of the PBA (black line). (**B**) The EDS measurements of surface C/O ratios of the blank control paper and papers coated with 5, 10 and 15% of PBA, respectively. (**C**) XPS survey spectra of the blank control paper and the PBA-coated paper (10%). XPS curve fitting of the C 1s photoelectron peak of blank control paper (**C1**) and PBA-coated paper (10%) (**C2**).

The ATR-FTIR analysis of blank control paper and PBA-coated paper, as well as the FTIR spectrum of PBA are shown in Figure 2A. After being coated with PBA (10%), new characteristic peaks of paper were found at 2950 and 1735 cm^−1^ due to the stretching vibration of –CH_3_ and C=O, respectively, which matched the FTIR characteristic peaks of PBA and were not characteristic peaks of cellulose. Compared with blank control paper, the ATR-FTIR spectrum of PBA-coated paper (10%) showed apparent decreases of –OH stretching vibration. These results proved the presence of PBA on the paper. 

The PBA coating was measured by EDS (Figure 2B). Since PBA has a large C_10_ carbon group, the surface carbon content of paper should increase with the increase of PBA’s concentration. Compared with the surface C/O ratio of 1.05 (C: 51.22 at %, O: 48.78 at %) for blank control paper, the ratios of papers coated with 5, 10 and 15% of PBA were increased to 3.02 (C: 75.13 at %, O: 24.87 at %), 4.31 (C: 81.16 at %, O: 18.84 at %) and 4.87 (C: 82.96 at %, O: 17.04 at %), respectively.

XPS analysis was also carried out in order to further detect the changes of surface elements’ composition and functional groups between the blank paper and the PBA-coated paper (10%). As reported in Figure 2C, after coating, the C/O ratio of the PBA-coated paper (10%) was increased significantly compared with that of the blank control paper, which was consistent with the results of EDS as mentioned above. The results of the XPS curve fitting of the C 1s photoelectron peak of the blank control paper and the PBA-coated paper are shown in Figure 2C1,C2. On both surfaces of the blank printer paper and the PBA-coated paper (10%), the C 1s signal can be deconvoluted by three components, corresponding to the C–C/C–H, C–O, O–C–O bonding states. For the PBA-coated paper sample, the C 1s signal showed a new peak located at 288 eV that corresponds to the C=O groups of PBA. All of these results can be attributed to the successful coating of PBA onto the paper surface.

### 3.3. Antifungal Colonization Assay

**Figure 3:**
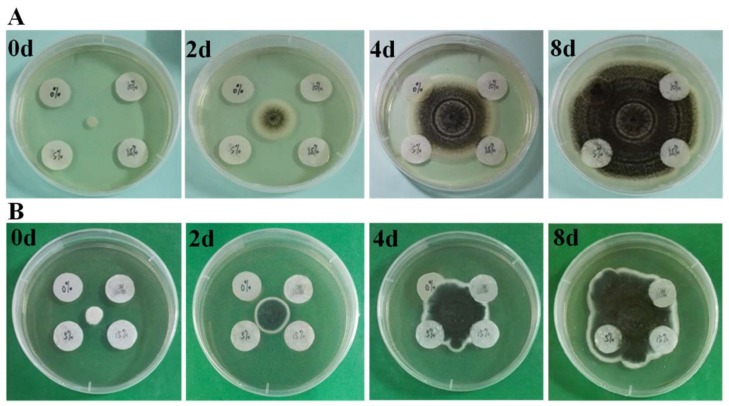
Antifungal effect of papers coated with 0 (left up), 5 (left down), 10 (right up) and 15% (right down) of PBA after incubating with (**A**) *A. niger* and (**B**) *Penicillium* sp. for eight days.

PBA and its copolymers have an outstanding antibacterial activity against Gram-negative and Gram-positive bacteria [23]. However, antifungal capability of PBA-based complexes has not been explored sufficiently. Therefore, the antifungal activity of PBA-coated paper was tested against *A. niger* and *Penicillium* sp. As shown in Figure 3, fungi began to spread from the center to the edge of the material. After four days of incubation, fungi mycelium began to climb the blank control paper. In contrast, PBA-coated paper began to form the inhibition edge of fungal growth. After eight days of incubation, fungal spores had spread over almost the entire surface of the blank control paper, while only a few cells were found on the PBA-coated paper surface. The antifungal capability improved with the increasing content of PBA. On the surface of the paper coated with 5% of PBA could also be found a few trace of fungal spores, while only scattered spores were found on papers coated with 10 and 15% of PBA. The sporadic spores on papers coated with 10 and 15% of PBA could not grow as they would on untreated paper. According to these results, the coating concentration of 10% was sufficient to ensure the antifungal capacity of PBA-coated paper.

**Figure 4:**
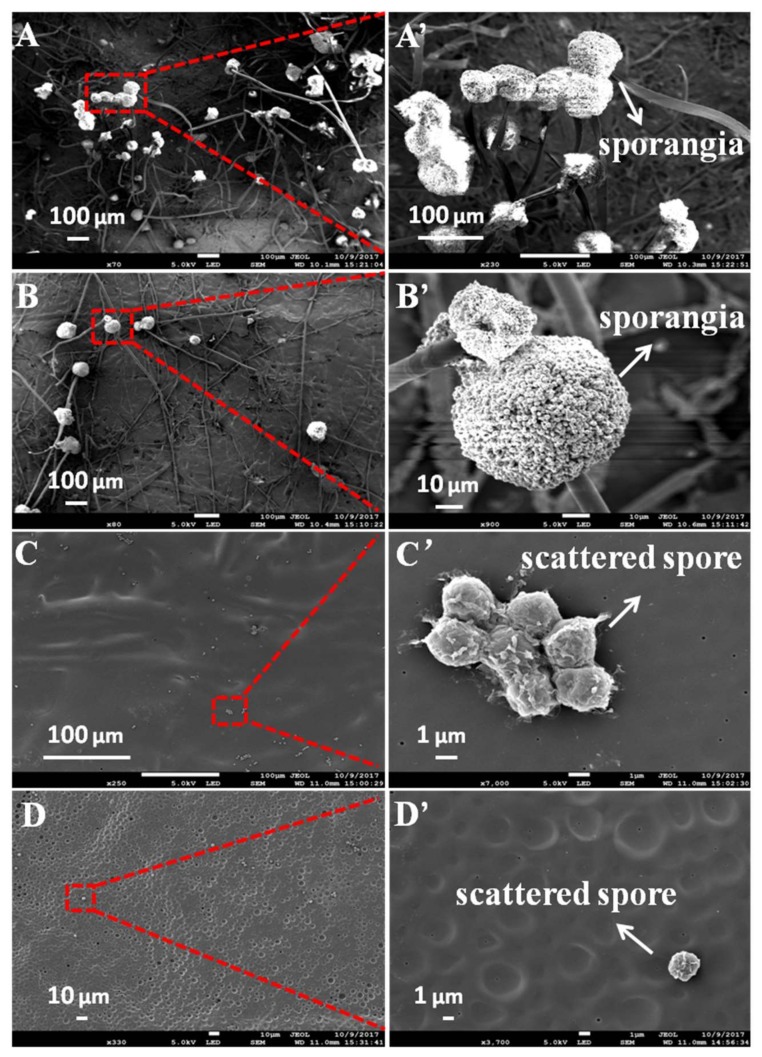
SEM images of *A. niger* cells on papers coated with 0 (**A**,**A’**), 5 (**B**,**B’**), 10 (**C**,**C’**) and 15% (**D**,**D’**) of PBA after incubating for eight days. The images of (**A’**–**D’**) show zoomed-in views of areas in the corresponding images of (**A**–**D**).

SEM characterization further demonstrated the distinct fungal morphology on the surfaces of blank control paper and paper coated with 5, 10 and 15% of PBA. As shown in Figure 4A, a large amount of *A. niger* sporangia and hypha were found on the surface of the blank control paper. From the inset image at high magnifications, we can see intact and lively sporangia. In contrast, the quantity of *A. niger* sporangia and hypha on the paper coated with 5% of PBA was significantly reduced (Figure 4B). Finally, almost no sporangia or hypha were found and only a few scattered spores could be found on the surface of paper coated with 10 and 15% of PBA (Figure 4C,D). These results further proved the above speculation: paper coated with PBA had good antifungal activity, and the coating concentration of 10% was sufficient to ensure the antifungal capacity. We deduced that physical falling instead of normal growth of the scattered spores happened on papers coated with 10 and 15% of PBA.

### 3.4. Anti-Spore Germination Evaluation

**Figure 5:**
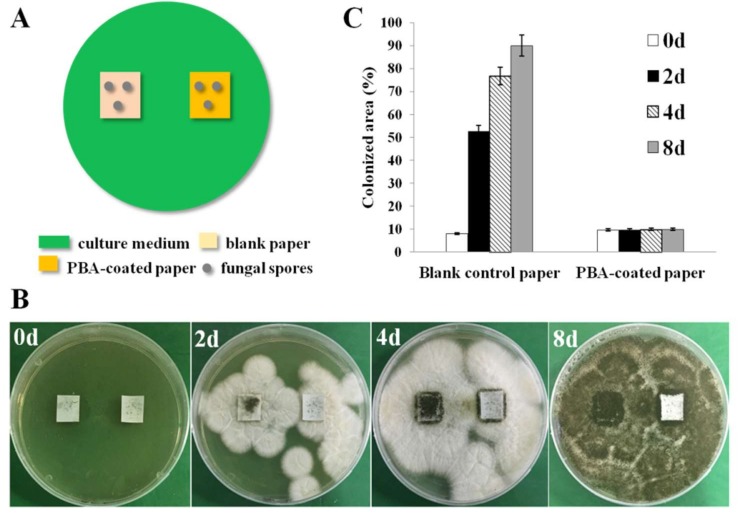
Antifungal evaluation under severe top-fouling conditions. (**A**) Schematic representation of the antifungal model. (**B**) Pictures of the antifungal adhesion effect against *A. niger* for eight days. (**C**) Fungal average colonized area after 0, 2, 4 and 8 days of incubation.

Fungi can easily colonize the surfaces of most materials and rapidly spread fungal spores. The combination of the high bio-receptivity of paper with inappropriate storage conditions or water-related emergency circumstances makes this material very susceptible to microbial deterioration, mainly fungi [7]. Two antifungal evaluation models in petri dishes were designed to simulate the conditions in which paper was contaminated during use and inappropriate storage. As shown in Figure 5A, the growth of fungal spores placed on the surface of the blank control paper and the PBA-coated paper (10%) was observed. After two days of incubation (Figure 5B), fungi grew normally on the surface of the blank control paper, and the colonized area was about 52.74% (Figure 5C). The area of fungal colony expanded with the increasing incubation time. After eight days of incubation (Figure 5B), the fungal colonized area on the blank control paper expanded to about 90.11% (Figure 5C). In contrast, the fungal colonized area on the PBA-coated paper stayed in the initial state (Figure 5C). The fungal spores placed on the surface of PBA-coated paper could not continue to grow normally, that is, the PBA-coated paper could inhibit the spore germination and the subsequent growth of fungi.

**Figure 6:**
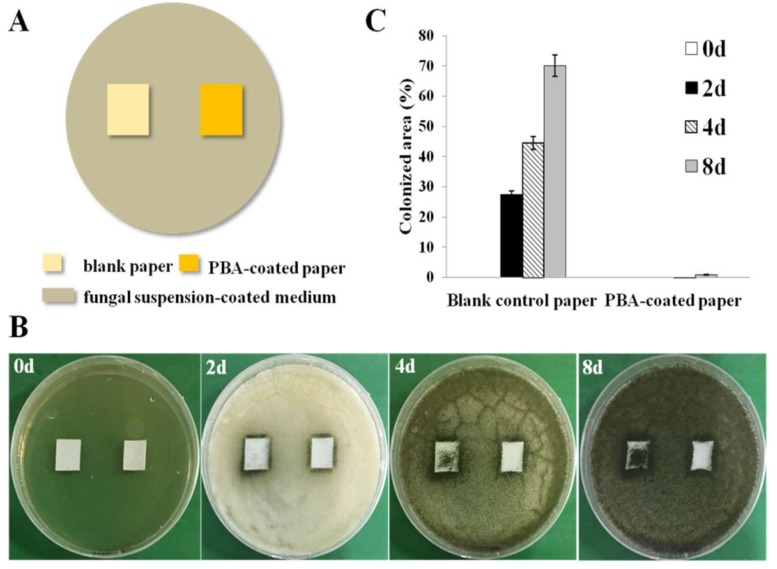
Antifungal evaluation under severe base-fouling conditions. (**A**) Schematic representation of the antifungal model. (**B**) Pictures of the antifungal adhesion effect against *A. niger* for eight days. (**C**) Fungal average colonized area after 0, 2, 4 and 8 days of incubation.

The second model is shown in Figure 6A, the samples (blank control paper and 10% PBA-coated paper) were put onto the plate coated with fungal suspension. The initial areas of fungi were 0% both of the blank control paper and the PBA-coated paper. After two days of incubation (Figure 6B), fungi began to climb onto the blank control paper. The fungal colonized areas of the blank control paper were about 27.33, 44.46 and 70.02% after 2, 4 and 8 days of incubation, respectively (Figure 6C). That is, the blank control paper was very susceptible to fungal contamination. By contrast, the surface of the PBA-coated paper remained clean even after eight days of incubation. These phenomena can be attributed to the excellent antifungal performance of the PBA-coated paper.

### 3.5. Physicochemical Properties Assay

The antifungal coating should not have a detrimental effect on paper’s inherent properties, such as brightness, water CA, pH and tensile strength [3,27,28]. Changes in paper physicochemical properties after coating with different concentrations of PBA were evaluated, and the results are shown in Table 1.

The optical property (brightness) is considered essential as far as printing and book papers are concerned [27]. We have known that the addition of Ag nanoparticles gives a yellowish tinge to the paper, thereby affecting its brightness [25]. However, the brightness of paper coated with 10% of PBA changed less than 5% according to the data.

The water contact angle of the blank control paper was about 59°. After coating with 5, 10 and 15% of PBA, the contact angles increased to 79°, 80° and 82°, respectively. That is, the hydrophobic property of the PBA-coated paper was improved [29].

pH is one measure of paper’s stability, as it indicates the degradation of α-cellulose and affects the printing of paper. There is a standard requirement of neutral pH (7.0) for writing and printing papers (BISIS:1848, 2007) [30]. The results indicated that the PBA coating did not cause any immediate alteration of pH on the paper samples, statistically.

After coating treatment, the mechanical property (tensile strength) of the paper samples declined. However, with the increasing coating concentration of PBA, the tensile strength of PBA-coated paper increased. The tensile strength of paper coated with 10% of PBA was similar to that of the control according to the data.

### 3.6. Inking Performance Assay

**Figure 7:**
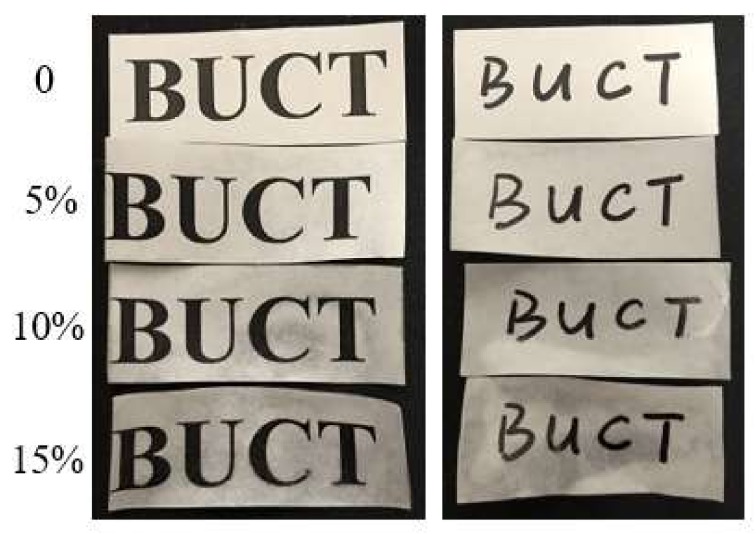
Pictures of printing and handwriting on PBA-coated paper after four months. The coating concentration was 0, 5, 10 and 15%, respectively, from the top to the bottom.

The inking performance was evaluated by visual observation on both printing and handwriting samples for four months. As shown in Figure 7, the clarity of ink printing and handwriting had no obvious difference after four months, which were both legible.

Inking performance was also evaluated through colorimetric measurement [1]. We printed the same black color block onto samples and compared the color difference between PBA-coated paper and blank control paper. As shown in Table 2, the total color difference value (ΔE) of PBA-coated paper (5, 10 and 15%) compared to the blank control paper was 0.51, 1.68 and 3.16, respectively. According to the instructions, a ΔE value less than one means a slight color difference, which is good for various applications. A ΔE value between one and two means a moderate color difference, which is also acceptable in most applications, for example printing. Therefore, the inking performance of the PBA coating (below 10%) is acceptable in general applications.

### 3.7. Mechanism Discussion

PBA is hydrophobic because BA has a large hydrophobic carbon cage structure [24]. When the coating concentration is increased, the water CA becomes hydrophobic gradually, as mentioned above. All of these results are clear indications of the successful coating of paper with PBA and the effectiveness of this coating method. Meanwhile, the water CA of PBA-coated paper was lower than that of pure PBAs [23,24]. This indicated that the PBA coating on papers did not form a dense film. As shown in Figure 1, the sprayed PBA solution was able to permeate downwards (~20.0 μm) and then dried, leaving some of the cellulose exposed. As a consequence, the antifungal adhesion effect is related to the concentration of PBA, where the coating concentration of 10% is perfect to ensure the antifungal capacity. It is worth noting that hydrophobicity may be helpful for antifungal adhesion, but it is not the main factor. It has been proven that the influence of surface hydrophobicity on the antifungal adhesion property is not a simple positive correlation [31]. PBAs that possess pendants of camphane-type bicyclic structures have complex carbon stereochemistry, which is more important than surface hydrophobicity on the antifungal performance of the PBA-coated paper.

## 4. Conclusions

In conclusion, we have successfully applied PBA polymers as antifungal coatings on paper by the spraying method, which is simple and efficient. The PBA-coated paper has outstanding antifungal performance against *A. niger* and *Penicillium* sp. under culture conditions for more than eight days. The coating concentration of 10% is proven to be the best manufacturing condition to ensure the antifungal capacity. The PBA coating displayed less influence on paper’s physicochemical properties including whiteness, pH and mechanical strength, and the resulting paper demonstrated good inking performance. The PBA-coated paper does not provide microbial resistance because it utilizes an advanced antimicrobial strategy of surface stereochemistry, reducing microbes’ adhesion other than killing microbes. Therefore, the PBA-coated paper presents great potential for wide applications of paper products.

## Figures and Tables

**Table 1:** The brightness, water CA, pH and tensile strength of the blank control paper and the papers coated with 5, 10 and 15% of PBA, respectively.

Concentration of PBA (%)	Brightness (%)	Water CA	pH	Tensile Strength (kN/m)
0	88.47 ± 0.07	59.4 ± 1.0°	7.23 ± 0.03	4.4 ± 0.2
5	86.49 ± 0.06	79.1 ± 2.0°	7.18 ± 0.02	3.8 ± 0.1
10	85.70 ± 0.10	80.3 ± 1.3°	7.12 ± 0.01	4.0 ± 0.1
15	82.85 ± 0.12	82.0 ± 1.6°	7.09 ± 0.02	4.3 ± 0.2

**Table 2:** Color difference values of blank control paper and paper coated with 5, 10 and 15% of PBA. Each value was an average of three replicates.

Concentration of PBA (%)	L*	a*	b*	ΔL*	Δa*	Δb*	ΔE*
0	15.41	0.73	0.46				
5	14.90	0.78	0.50	−0.51	0.05	0.04	0.51
10	13.75	0.81	0.68	−1.66	0.08	0.22	1.68
15	12.25	0.79	0.48	−3.16	0.06	0.02	3.16
